# Non-invasive Oscillometry-Based Estimation of Cardiac Output – Can We Use It in Clinical Practice?

**DOI:** 10.3389/fphys.2021.704425

**Published:** 2021-08-03

**Authors:** Alexander Reshetnik, Jonida Gjolli, Markus van der Giet, Friederike Compton

**Affiliations:** Department of Nephrology and Intensive Care Medicine, Corporate Member of Freie Universität Berlin, Humboldt-Universität zu Berlin, Charité – Universitätsmedizin Berlin, Berlin, Germany

**Keywords:** non-invasive, cardiac output measurement, thermodilution, oscillometric, pulse wave analysis

## Abstract

While invasive thermodilution techniques remain the reference methods for cardiac output (CO) measurement, there is a currently unmet need for non-invasive techniques to simplify CO determination, reduce complications related to invasive procedures required for indicator dilution CO measurement, and expand the application field toward emergency room, non-intensive care, or outpatient settings. We evaluated the performance of a non-invasive oscillometry-based CO estimation method compared to transpulmonary thermodilution. To assess agreement between the devices, we used Bland–Altman analysis. Four-quadrant plot analysis was used to visualize the ability of Mobil-O-Graph (MG) to track CO changes after a fluid challenge. Trending analysis of CO trajectories was used to compare MG and PiCCO^®^ calibrated pulse wave analysis over time (6 h). We included 40 patients from the medical intensive care unit at the Charité – Universitätsmedizin Berlin, Campus Benjamin Franklin between November 2019 and June 2020. The median age was 73 years. Forty percent of the study population was male; 98% was ventilator-dependent and 75% vasopressor-dependent at study entry. The mean of the observed differences for the cardiac output index (COI) was 0.7 l^∗^min^–1*^m^–2^ and the lower, and upper 95% limits of agreement (LOA) were -1.9 and 3.3 l^∗^min^–1*^m^–2^, respectively. The 95% confidence interval for the LOA was ± 0.26 l^∗^min^–1*^m^–2^, the percentage error 83.6%. We observed concordant changes in CO with MG and PiCCO^®^ in 50% of the measurements after a fluid challenge and over the course of 6 h. Cardiac output calculation with a novel oscillometry-based pulse wave analysis method is feasible and replicable in critically ill patients. However, we did not find clinically applicable agreement between MG and thermodilution or calibrated pulse wave analysis, respectively, assessed with established evaluation routine using the Bland–Altman approach and with trending analysis methods. In summary, we do not recommend the use of this method in critically ill patients at this time. As the basic approach is promising and the CO determination with MG very simple to perform, further studies should be undertaken both in hemodynamically stable patients, and in the critical care setting to allow additional adjustments of the underlying algorithm for CO estimation with MG.

## Introduction

Cardiac output (CO) is a key determinant of oxygen delivery and thus an important parameter to assess the hemodynamic situation of critically ill patients, guide perioperative goal-directed therapy, and monitor response to therapeutic interventions (Cecconi et al., 2014). CO can be measured or estimated using invasive, minimally invasive, and non-invasive techniques as well (Sakka et al., 1999; Saugel et al., 2021). While invasive indicator dilution techniques such as pulmonary arterial or transpulmonary thermodilution remain the clinical gold standard and reference methods for CO measurement, and minimally invasive CO determination methods are also available for use in the intensive care unit (ICU), there is a currently unmet need for non-invasive techniques to further simplify CO estimation, reduce complications related to invasive techniques such as pulmonary artery catheterization, and to facilitate use in non-intensive care settings, e.g., for rapid hemodynamic assessment in the emergency room or even in outpatient settings.

In this study, we evaluated a non-invasive oscillometry-based pulse wave analysis CO estimation technique for use in critically ill patients.

## Materials and Methods

Eligible patients treated in the medical ICU of the Charité – Universitätsmedizin Berlin, Campus Benjamin Franklin were enrolled between November 2019 and June 2020. The Charité – Universitätsmedizin Berlin regional research ethics committee approved the study (ref: EA1/184/15). All methods were performed following the relevant guidelines and regulations.

Only patients monitored hemodynamically with the invasive CO determination PiCCO^®^ device (Pulsion Medical Systems, Feldkirchen, Germany) as part of their ICU treatment were included in the study. Exclusion criteria were age below 18 years, pregnancy, known severe aortic valve, aortic arch, axillary, or brachial artery stenosis, as well as cardiac arrhythmias precluding non-invasive calculation of haemodynamic parameters. Patients were considered haemodynamically unstable if mean arterial pressure (MAP) was <65 mmHg or vasopressor therapy was necessary to maintain MAP ≥ 65 mmHg.

The test device for non-invasive CO estimation was the Mobil-O-Graph (MG)^®^ (MG; I.E.M., Stolberg, Germany) blood pressure monitoring device equipped with an improved CO calculation algorithm (Hypertension Management Software Client 5.2, 2018). With MG, CO is estimated from the arterial pulse wave derived with a high fidelity pressure sensor integrated into the blood pressure cuff while being inflated at diastolic blood pressure level for 10 s. Cuff size was chosen according to manufacturer instructions: regular size for arm circumferences of 24–34 cm, large size for arm circumference 32–42 cm. The measurement site was left or right upper arm. The construction of the aortic pressure waveform was made using generalized transfer functions (Fourier analysis and decompensation into wave harmonics) and transformation from aortic pressure to aortic flow waveform was performed with an adopted, and multidimensional Windkessel model. Basic principle used is derivation of stroke volume (SV) from pulse contour analysis (PCA) determined with oscillometry. SV is proportional to the area of the flow curve during systole. The computation of the aortic flow from pressure was based on the 3-element windkessel model determined by aortic characteristic impedance, aortic compliance and peripheral resistance. These parameters were identified using Levenberg–Marquardt algorithm. SV was derived from the time lag between aortic pressure and flow curve (characteristic impedance, Zc). Finally, CO was calculated by multiplying SV with the heart rate. Detailed underlying mathematical principles related to CO estimation with MG are described elsewhere (Wassertheurer et al., 2008). For comparison with reference CO, the mean value of two consecutive CO determinations performed with MG was used in each patient. CO was corrected for body surface area and expressed as cardiac output index (COI).

Reference CO was measured using transpulmonary thermodilution with the PiCCO^®^ system: A bolus of 20 mL of cold (0–6°C) 5% glucose solution was manually injected (injection time ≤ 10 s) into the distal lumen of a central venous catheter while the patient was in a supine position and then detected in the systemic circulation by a thermistor-tipped femoral artery catheter (Pulsiocath PV2015L20, Pulsion Medical Systems, Feldkirchen, Germany). As with MG CO estimation, two consecutive CO measurements were performed, a mean was calculated for inclusion in the final analysis and COI calculated.

We performed CO measurements with the test (MG) and reference (thermodilution with PiCCO^®^) devices before, and after a fluid challenge with 150 mL of crystalloid solution. In addition, we performed trend analyses with MG and PiCCO^®^ -calibrated pulse wave analysis over a maximum of 6 h (Laight and Levin, 2015). Furthermore, invasively and non-invasively measured blood pressure and heart rate were obtained from routine hemodynamic monitoring and included in the comparison analysis. Demographic and specific clinical patient characteristics were obtained from the hospital patient data management systems.

### Statistics

Data were analyzed using Graph-Pad Prism 5 (GraphPad Software, La Jolla, CA, United States) and SPSS Statistics 25.0 (IBM, New York, NY, United States). Continuous variables are presented as median with quartiles. Categorical variables are presented as absolute numbers with percentages. Statistical differences between paired measurements were assessed using a nonparametric Wilcoxon test, and a two-sided *p*-value of <0.05 was regarded as statistically significant. Bland–Altman analysis was used to assess agreement between test and reference device where the mean of the observed differences was calculated as a measure for accuracy and the 95% LOA as a measure for precision including their 95% confidence intervals (Bland and Altman, 2007; Lu et al., 2016). Furthermore, we calculated the percentage error of agreement, which was computed from the one-sided width of the LOA divided by the average of CO. For trending analysis after the fluid challenge, the 4-quadrant plot technique was used. We expressed CO change after fluid challenge as relative change according to the following equation:

$$\frac{COafterfluidchallenge-CObeforefluidchallenge}{CObevorefluidchallenge}*100\%$$

For the purpose of the study, we defined an exclusion zone of 15% (Saugel et al., 2015). To compare long-term trends in CO, slopes of the trajectories for each method were calculated as shown in the Supplementary Figures 1, 2. Within-subject method reliability was assessed with intraclass correlation coefficient calculated with the two-way mixed model.

## Results

We included 45 participants in the study. Five patients had incomplete measurement data sets due to technical problems so that 40 patients were included in the final analysis. The median age was 73 years, 40% of the study population was male, 98% was ventilator-dependent, and 75% vasopressor-dependent at study entry. Clinical and demographic parameters are presented in Table 1. Concerning hemodynamic data, there were significant differences not only between CO determinations with MG and PiCCO^®^, respectively, but also between non-invasive and invasive blood pressure measurements: Both diastolic and MAPs were significantly higher with MG than measured invasively in the femoral artery (70 vs. 55 mmHg, *p* < 0,001, and 90 vs. 80 mmHg, *p* < 0,001, respectively). Conversely, systolic blood pressure was lower with MG than with invasive measurement (118 vs. 123 mmHg), even though this difference was not statistically significant. Both CO and COI were significantly lower with MG as compared with PiCCO^®^ (4.9 vs. 6.1 l^∗^min^–1^ and 2.6 vs. 3.2 l^∗^min^–1*^m^–2^, respectively). Obtained CO values ranged from 3.2 to 10.7 l^∗^min^–1^ for MG and from 2.9 to 17.8 l^∗^min^–1^ for PiCCO^®^. Further details concerning hemodynamic parameters are depicted in Table 2.

**TABLE 1:** Baseline patient characteristics.

Age, years	73 (62;82)
Male	16 (40%)
Height, cm	170 (165;180)
Weight, kg	74 (65;89)
Body surface area, m^2^	1,90 (1,68;2,09)
Body mass index, kg/m^2^	25,5 (22,5;27,8)
SAPS II^#^-Score at study entry	66 (51;77)
SOFA*-Score at study entry	11 (8;13)
Vasopressor therapy at study entry, n (%)	30 (75%)
Ventilation	
On intensive care unit admission	29 (73%)
At study entry	39 (98%)
Duration of intensive care unit stay, days	22 (11;32)
Mortality	
28-days mortality	18 (45%)
3 months mortality	24 (60%)
Chronic illness	36 (90%)
Coronary heart disease	14 (35%)
Prior myocardial infarction	9 (23%)
Chronic heart failure	9 (23%)
Arterial hypertension	30 (75%)
Smoker	10 (25%)
Dyslipidemia	11 (28%)
Diabetes mellitus	19 (48%)
Peripheral arterial disease	5 (13%)
Prior stroke	11 (28%)
Acute kidney injury	24 (60%)
Dialysis dependent kidney injury	17 (43%)
Chronic kidney disease	9 (23%)
Sinus rhythm at study entry	27 (68%)

*Continuous parameters presented as median (quartiles), categorical variables presented as number of subjects (%).*

**TABLE 2:** Hemodynamic parameters with Mobil-O-Graph^®^ and PiCCO^®^.

	Mobil-O-Graph^®^	PiCCO^®^	*p*-Value*
Systolic blood pressure, mmHg	118 (109;131)	123 (111;137)	0.658
Diastolic blood pressure, mmHg	70 (63;78)	55 (50;64)	<0.001
Mean arterial pressure, mmHg	90 (84;102)	80 (70;87)	<0.001
Heart rate, 1/min	89 (73;99)	87 (74;99)	0.6
Cardiac output, l*min^–1^	4.9 (4.2;5.8)	6.1 (5.0;7.5)	<0.001
Cardiac output index, l*min^–1^*m^–2^	2.6 (2.2;3.3)	3.2 (2.7;4.0)	<0.001

Parameters presented as median (quartiles). *Wilcoxon–Test for related samples.

**FIGURE 1:**
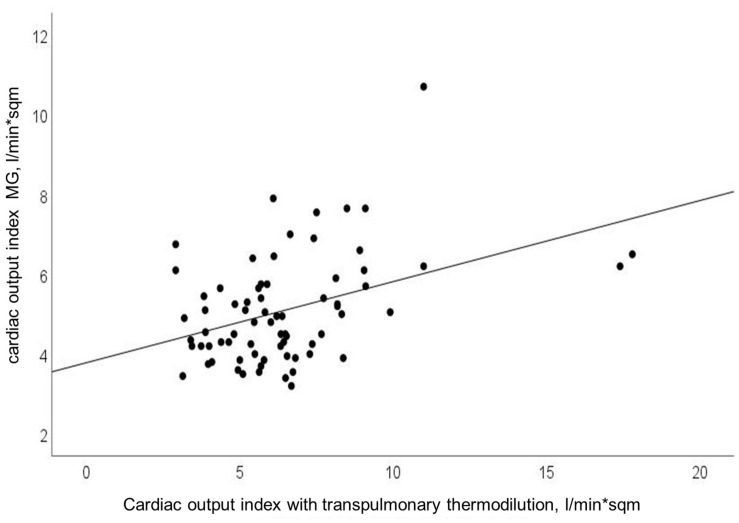
Relationship between cardiac output index (CO-Index) measured with transpulmonary thermodilution and Mobil-O-Graph (MG); Pearson’s *r* = 0.40; *p* < 0.0001.

Cardiac output and COI obtained with MG significantly correlated with CO and COI obtained with reference device (Pearson’s *r* = 0.40; *p* < 0.0001). Findings for COI are presented in Figure 1. Using explorative data analysis, we identified four values as “outliers.” We performed addition correlation analysis with Pearson’s *r* = 0.28 (*p* = 0.02), which is shown in Supplementary Figure 2.

**FIGURE 2:**
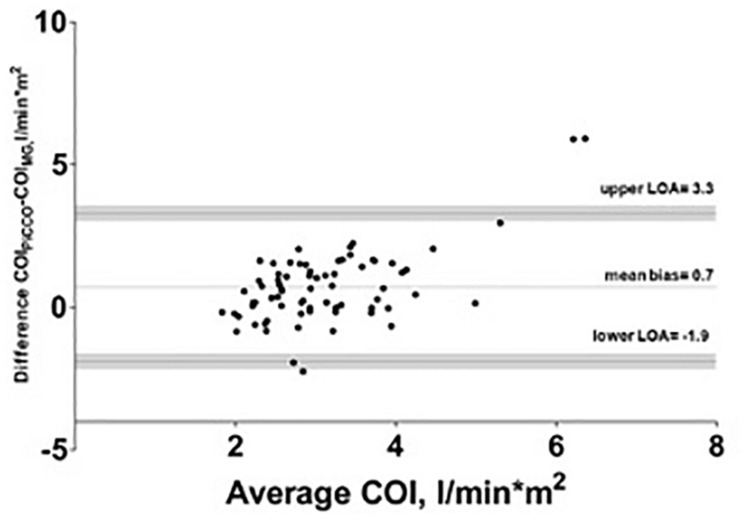
Bland–Altman analysis of cardiac output index (COI) with test (Mobil-O-Graph-MG) and reference method (PiCCO^®^ thermodilution). Gray shaded area represents 95% confidence interval of limits of agreement (LOA).

The mean of the observed differences for the CO was 1.3 l^∗^min^–1^ and the lower, and upper 95% LOA were -3.5 and 6.1 l^∗^min^–1^, respectively. 95% confidence interval for the LOA was ± 0.49 l^∗^min^–1^. The mean of the observed differences for the COI was 0.7 l^∗^min^–1*^m^–2^ and the lower, and upper 95% limit of agreement is -1.9 and 3.3 l^∗^min^–1*^m^–2^, respectively. 95% confidence interval for the LOA was ± 0.26 l^∗^min^–1*^m^–2^ (Figure 2). The percentage error was 83.6%.

The intraclass correlation coefficient for PiCCO^®^ was 0.97 and for MG 0.89.

**FIGURE 3:**
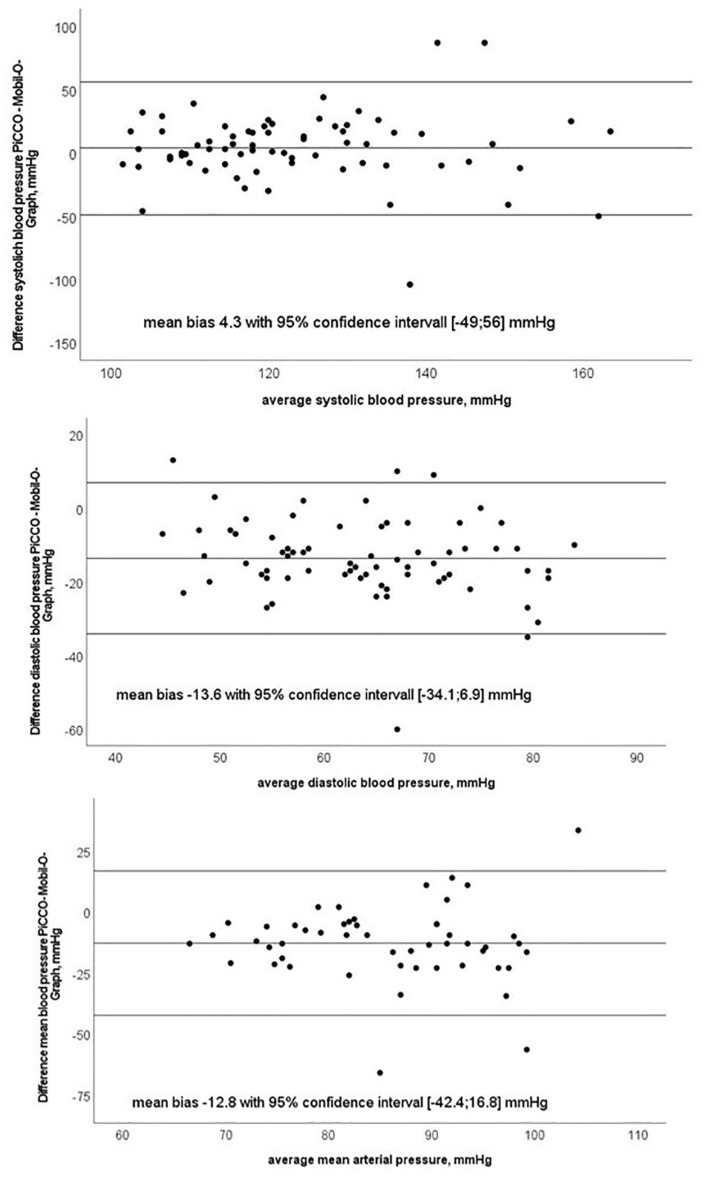
Bland–Altman plots of systolic, diastolic and mean blood pressure with test (Mobil-O-Graph-MG) and reference method (PiCCO^®^ thermodilution).

Bland–Altman plots for systolic, mean, diastolic BP are shown in Figure 3.

**FIGURE 4:**
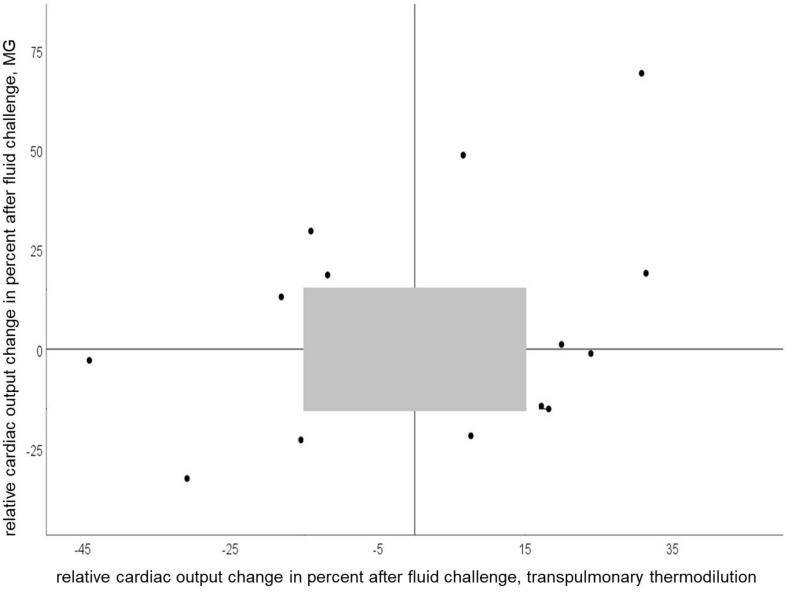
Four-quadrant plot of relative cardiac output change after a fluid challenge of 150 ml with Mobil-O-Graph and PiCCO^®^ (concordance rate = 50%). Gray shaded area represents exclusion zone of 15%.

We observed concordant changes in COI after fluid challenge in 50 percent of all measurements. Figure 4 shows relative CO change (per cent) after fluid challenge for the test and reference device.

In the trending analysis of calculated CO with MG and PiCCO^®^ over 6 h, we observed concordant increases or decreases in the CO slope in 50 percent of measurements as a correlate for the trend of changes.

## Discussion

In the present study, we evaluated the performance of a non-invasive oscillometry-based method for CO determination (MG) under static conditions and its ability to trend the changes in CO after fluid challenge and over a course of several hours in critically ill patients. We found a moderate correlation between CO estimation with MG and reference PiCCO^®^ measurements and an acceptable mean bias between test and reference device with wide margins in LOA and a high percentage error. Since we performed repeated measurements per subject, high LOA and percentage error may be partly caused by the imprecision of either method (Saugel et al., 2020). We, thus, performed reliability analysis for the test and reference methods, respectively, and observed excellent intraclass correlation coefficients indicating low within-subject variance and high reliability of both methods. The algorithm used in MG relies on the precise derivation of the arterial pressure curve. Indeed, the exact determination of the arterial pressure curve with MG is a challenging process as data acquisition is realized using an occlusive cuff applied to the upper arm recording a wide range of oscillometric amplitudes (Wassertheurer et al., 2008). In our study cohort consisting of critically ill and vasopressor-dependent patients, the arterial pressure waveform signal in the brachial artery can be disturbed or differ significantly from that in the femoral artery (Teboul et al., 2016). Usually, diastolic and mean blood pressure are similar in peripheral and central arteries, and systolic blood pressure is higher in the femoral compared to brachial artery due to wave reflection (Kroeker and Wood, 1955). However, we observed clinically relevant lower systolic and clinically relevant higher diastolic and MAP with even statistical significance for diastolic and MAP between MG and PiCCO^®^, which is a common finding when blood pressure levels between peripheral (non-invasive), and central (invasive) measurements have been compared in critically ill patients. Imprecise estimation of central arterial pressure during hemodynamic instability can influence the accuracy of derived CO (Compton et al., 2008; Compton and Schafer, 2009).

Plenty of non-invasive CO monitoring devices, which rely on non-invasive pulse wave analysis, pulse wave transit time, or thoracic bioimpedance as a basic principle for CO calculation, have been described in the literature (Papaioannou et al., 2020). Acknowledging that direct comparison with other non-invasive CO determination methods based only on published literature has its limitations due to different reference methods used and different study populations, our data ranged within the margins of published evidence. However, a very recent review on currently available technologies for CO determination using pulse wave analysis does not mention oscillometry as a potential method (Saugel et al., 2021), even though it has some very practical advantages like easiness, and rapidity of measurements as well as no requirement for any specific operator training.

Algorithmic refinements had been made by the manufacturer in 2018. Compared to a previous study with the older software we observed a lower mean bias with comparable LOA and higher percentage error in this study (Reshetnik et al., 2017). A recent study by Papaioannou et al. (2020) showed a comparable mean bias in a smaller cohort in the ICU. According to the cut-off of 30 percent for the percentage error, proposed by Critchley and Critchley (1999) the percentage error found in our study points to precision in need for improvement. As visualized in Figures 1, 2, we observed some extreme values, which can be considered as outliers. Additional correlation analysis without outliers showed same finding of poor correlation between test and reference device.

In the clinical setting tracking CO changes with therapy or overtime is usually more important than the determination of single absolute CO values. Our study is one of the few, in which not only CO response to a singular fluid challenge was considered, but also the ability to track CO changes over a longer period. We observed concordant changes in CO with MG and PiCCO^®^ in 50 percent of the measurements after volume change and over several hours, which points to a weak concordance. Of note, in 67% of cases with non-concordant CO changes recordings were performed while the patients received high-dose vasopressor therapy. Similar findings were reported for other CO calculation methods (Monnet et al., 2010). Indeed, there is evidence that pulse wave analysis devices may struggle to adapt to changes in vascular tone induced by vasopressors (Meng et al., 2011).

We acknowledge several limitations of the study. The number of enrolled subjects was relatively small. We did not use pulmonary artery catheter as the reference method. The vasopressor doses possibly contributing to significant variation in calculated CO varied between the subjects. Arterial compliance has a major influence on CO estimation derived from pulse wave analysis. Rapid changes in vasomotor tone, in particular with higher vasopressor dose, can have an impact on arterial compliance and consequently impair the CO estimation. Due to the non-randomized study design, we cannot account for all possible confounding factors, which may influence the CO. We did not compare BP values between both arms prior to the validation.

## Conclusion

To conclude that CO calculation with a novel oscillometry-based pulse wave analysis method is feasible and replicable in critically ill patients. However, a clinically applicable agreement between MG and thermodilution used as a reference method in critically ill patients was not observed using the Bland–Altman approach and with trending analysis methods. In summary, we do not recommend using this method in critically ill patients at this time. As the basic approach is promising and the CO calculation with MG very simple, further studies should be performed in hemodynamic stable patients and critical care setting to gain additional data to be able to further adjust the underlying algorithm for CO determination in this device.

## Data Availability Statement

The raw data supporting the conclusions of this article will be made available by the authors, without undue reservation.

## Ethics Statement

The studies involving human participants were reviewed and approved by Charité Ethics Committee. The patients/participants provided their written informed consent to participate in this study.

## Author Contributions

AR: conception of design, analysis and interpretation of the data, drafting the article, and final approval of the version to be published. JG: conception of design, analysis and interpretation of the data, and drafting the article. MG: conception of design and providing intellectual content of critical importance. FC: interpretation of the data, revising the article, and final approval of the version to be published. All authors contributed to the article and approved the submitted version.

## Conflict of Interest

The authors declare that the research was conducted in the absence of any commercial or financial relationships that could be construed as a potential conflict of interest.

## Publisher’s Note

All claims expressed in this article are solely those of the authors and do not necessarily represent those of their affiliated organizations, or those of the publisher, the editors and the reviewers. Any product that may be evaluated in this article, or claim that may be made by its manufacturer, is not guaranteed or endorsed by the publisher.

## Supplementary Material

Supplementary Figure 1 Cardiac output change in a patient over the time course registered with Mobil-O-Graph^®^ and PiCCO^®^ pulse contour analysis.

Supplementary Figure 2 Relationship between cardiac output index (CO-Index) measured with transpulmonary thermodilution and Mobil-O-Graph (MG) after exclusion of outliers; Pearson’s *r* = 0.28; *p* = 0.02.
