# Supplementary figures and images for: Non-invasive Oscillometry-Based Estimation of Cardiac Output – Can We Use It in Clinical Practice?
Source: Front Physiol. 2021 Aug 3;12:704425. doi: 10.3389/fphys.2021.704425 (PMC8369501; doi:10.3389/fphys.2021.704425)

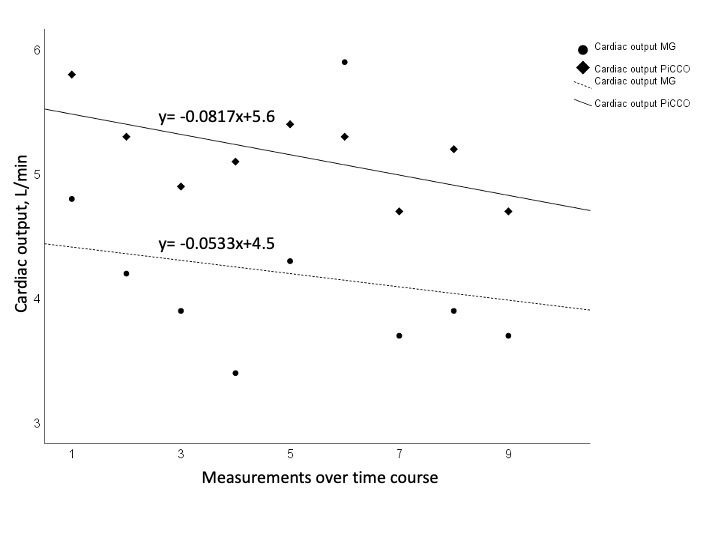

Supplement: Supplementary Figure 1 — Cardiac output change in a patient over the time course registered with Mobil-O-Graph® and PiCCO® pulse contour analysis. [file Image_1.JPEG]

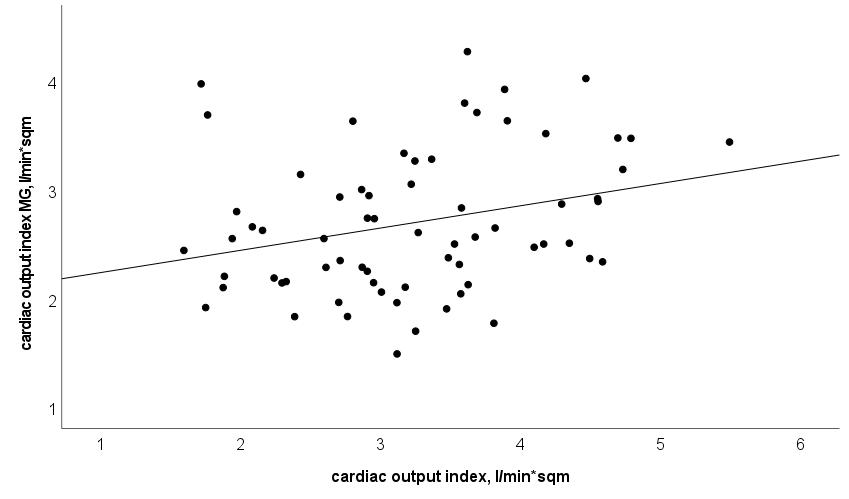

Supplement: Supplementary Figure 2 — Relationship between cardiac output index (CO-Index) measured with transpulmonary thermodilution and Mobil-O-Graph (MG) after exclusion of outliers; Pearson’s r = 0.28; p = 0.02. [file Image_2.JPEG]
